# Use of population pharmacokinetic‐pharmacodynamic modelling to inform antimalarial dose optimization in infants

**DOI:** 10.1111/bcp.16132

**Published:** 2024-06-10

**Authors:** Clifford G. Banda, Joel Tarning, Karen I. Barnes

**Affiliations:** ^1^ Malawi‐Liverpool‐Wellcome Programme Blantyre Malawi; ^2^ Division of Clinical Pharmacology, Department of Medicine University of Cape Town Cape Town South Africa; ^3^ Kamuzu University of Health Sciences (formerly College of Medicine and Kamuzu College of Nursing, University of Malawi) Blantyre Malawi; ^4^ Centre for Tropical Medicine and Global Health, Nuffield Department of Medicine University of Oxford Oxford UK; ^5^ Mahidol Oxford Tropical Medicine Research Unit, Faculty of Tropical Medicine Mahidol University Bangkok Thailand; ^6^ WorldWide Antimalarial Resistance Network (WWARN), Pharmacology Scientific Group University of Cape Town Cape Town South Africa

**Keywords:** antimalarials, infants, modelling, pharmacokinetic‐pharmacodynamics

## Abstract

Infants bear a significant malaria burden but are usually excluded from participating in early dose optimization studies that inform dosing regimens of antimalarial therapy. Unlike older children, infants' exclusion from early‐phase trials has resulted in limited evidence to guide accurate dosing of antimalarial treatment for uncomplicated malaria or malaria‐preventive treatment in this vulnerable population. Subsequently, doses used in infants are often extrapolated from older children or adults, with the potential for under‐ or overdosing. Population pharmacokinetic‐pharmacodynamic (PK‐PD) modelling, a quantitative methodology that applies mathematical and statistical techniques, can aid the design of clinical studies in infants that collect sparse pharmacokinetic data as well as support the analysis of such data to derive optimized antimalarial dosing in this complex and at‐risk yet understudied subpopulation. In this review, we reflect on what PK‐PD modelling can do in programmatic settings of most malaria‐endemic areas and how it can be used to inform antimalarial dose optimization for preventive and curative treatment of uncomplicated malaria in infants. We outline key developmental physiological changes that affect drug exposure in early life, the challenges of conducting dose optimization studies in infants, and examples of how PK‐PD modelling has previously informed antimalarial dose optimization in this subgroup. Additionally, we discuss the limitations and gaps of PK‐PD modelling when used for dose optimization in infants. To utilize modelling well, there is a need to generate useful, sparse, PK and PD data in this subpopulation to inform antimalarial optimal dosing in infancy.

## Graphical abstract

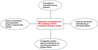

## 1 INTRODUCTION

Sub‐Saharan Africa bears more than 93% of morbidity and mortality due to *Plasmodium falciparum* malaria. In this setting, approximately 36% of the burden is experienced in infants.^1^, ^2^ After birth, an infant (a child <1 year of age) is protected from malaria due to the immunity conferred by the mother. Nevertheless, they become vulnerable, as early as 3 months of age, when this acquired immunity begins to wane.^3^ Subsequently, this puts infants at an increased risk of rapid disease progression, severe malaria and death. To minimize this burden, the World Health Organization (WHO) initially recommended the use of sulfadoxine‐pyrimethamine (SP), administered with routine health facility visits, for intermittent preventive treatment of malaria in infancy (IPTi) in areas of moderate to high malaria transmission in sub‐Saharan Africa.^4^ Recently, this recommendation has been extended beyond the first year of life and is now termed perennial malaria chemoprevention (PMC).^5^ However, although recommended by the WHO for over a decade, malaria chemopreventive treatment in infancy has had limited uptake by most malaria‐endemic countries, in part due to the reported increasing resistance of malaria parasites to SP. Fortunately, monthly administration of a newer antimalarial drug, dihydroartemisinin‐piperaquine (DP), an artemisinin‐based combination therapy (ACT), has shown higher protective efficacy than SP.^6^, ^7^ However, a monthly regimen for malaria chemoprevention is a challenge to implement in programmatic settings.^8^, ^9^, ^10^ While acknowledging the potential for ACTs, such as DP, for malaria chemoprevention in young children, the WHO has called for more evidence of their safety, efficacy and adherence to multi‐day regimens, and when administered during routine health facility visits.^11^ This highlights an urgent need for evidenced‐based antimalarial dosing recommendations in infants, particularly in those under 6 months of age, for both malaria treatment and chemopreventive treatment. This should be done together with other malaria‐preventive interventions such as vaccines and insecticide‐treated bed nets.

However, most therapeutic areas have excluded infants from participating in early dose optimization clinical trials due to ethical and logistical concerns.^12^ This is no different within the malaria field where most clinical trials to optimize the treatment (and prevention) of uncomplicated malaria have excluded infants. This has not been the case with older children, mostly above 1 year of age, with uncomplicated malaria who are usually studied in phase III and severe malaria studies. Consequently, antimalarial doses for the treatment of uncomplicated malaria have been extrapolated from older children as well as adults for use in infants. The challenge with such extrapolation is that antimalarial medications used for symptomatic treatment have the potential to be inaccurately dosed in infants when treating malaria.^13^ This may potentially result in efficacy or safety concerns. For example, the use of the current dispersible formulation of artemether‐lumefantrine (AL) (Coartem®) in infants weighing <5 kg resulted in a two‐ to three‐fold increase in artemether and its metabolite dihydroartemisinin compared with children weighing >5 kg.^14^ To address this challenge, the PAMAfrica consortium was established to develop a new fixed‐dose combination of AL for infants weighing <5 kg since the current AL dispersible formulation targeted children between 5 and 35 kg.^15^ While there are ethical and logistical challenges in conducting dose optimization studies in infants,^16^, ^17^ such as the intense frequency of blood sampling that is often required, it is imperative that subgroups that bear the largest burden of disease are included, as early as possible, in clinical trials that inform dosing regimens. Quantitative techniques, such as population pharmacokinetic‐pharmacodynamic (PK‐PD) modelling, have the advantage of supporting the design and analysis of data from studies that can be conducted to overcome the logistical hurdles associated with conducting dose optimization studies in infants.

In this review, we describe how PK‐PD modelling can be used to inform antimalarial dose optimization for preventive and curative treatment of uncomplicated malaria in infants. We outline key developmental physiological changes that may affect drug exposure in early life, the challenges of conducting dose optimization studies in infants, the role of PK‐PD modelling in informing antimalarial dose optimization processes and examples of how PK‐PD modelling has previously informed antimalarial dose optimization in infants. Additionally, we discuss the limitations and gaps of PK‐PD modelling when used for dose optimization in infants as well as other modelling techniques that can complement population PK‐PD methods for dose optimization in this subgroup.

## 2 DEVELOPMENTAL PHYSIOLOGICAL CHANGES IN EARLY LIFE AFFECT DRUG EXPOSURE

Infancy is characterized by rapid physiological changes that impact the pharmacokinetic exposure profiles of various drugs used during this period.^18^ Key pharmacokinetic changes in this subgroup include altered absorption, reduced distribution of drugs to sites of action, and reduction in drug metabolism because of developing metabolic processes (Table 1).^19^, ^20^ These changes are driven by differences in body size, body composition, enzyme maturation and end‐organ perfusion. Drugs may be affected by these changes to different extents, hence the need for PK data in infants, albeit sparse, to optimize their dosing. Infants are, therefore, a complex population that requires special attention to achieve optimal dosing of therapeutic agents such as antimalarial medications.

**TABLE 1:** Physiological changes in infancy that affect drug pharmacokinetics.

Pharmacokinetic process	Physiological change(s)
**Absorption**	Increased intestinal transit time Reduced gastric emptying: delayed in infants under 6–8 months and shorter in older infants Delayed/immature gastric transporter expression
**Distribution**	Age‐dependent body composition influencing the volume of distribution of drugs (including increased thickness, perfusion, hydration and body surface area in infants) Increased body water:fat ratio highest in neonates Plasma protein binding lower in neonates
**Metabolism**	Reduced hepatic metabolism in neonates (cytochrome P‐450 and glucuronosyltransferase isoforms) reaching adult levels by 6–12 months
**Elimination**	Elimination increases nonlinearly with weight Renal function reaches maturity at approximately 1–2 years of age

## 3 CHALLENGES OF DOSE OPTIMIZATION STUDIES IN INFANTS

Standard dose optimization studies involve administering a drug and collecting frequent blood samples thereafter, at prespecified time intervals, to derive individual pharmacokinetic primary parameters such as clearance and volume of distribution as well as secondary parameters such as the area under the concentration–time curve (AUC), peak concentration (*C*_max_) and terminal elimination half‐life (*t*_1/2_).^21^ Although such optimization studies provide detailed individual pharmacokinetic parameter estimates, which can be aggregated to provide information about a particular group of participants, they have several challenges when extrapolated to infants. First, the volume of blood and frequency of sampling that is acceptable to be collected from infants and very young children is limited. Guidelines indicate that this should not exceed 1% of total blood volume at any one time (0.8 mL/kg) or 3% (2.4 mL/kg) within 1 month.^22^ Such small volumes may be problematic for most pharmacokinetic assays,^23^ including those for antimalarials. However, great advances have been seen in recent years with the development of low‐volume LC–MS/MS assays for antimalarial drugs, such as dried blood on filter paper‐based assays, showing good sensitivity and accuracy.^24^, ^25^, ^26^ Second, the logistics of collecting multiple capillary samples still requires infants to remain longer at the health facility or return frequently. This may not always be acceptable to primary caregivers.^21^ Third, there is the ethical concern of the risk–benefit of each infant's study participation. Ethically acceptable studies are those that pose minimal risk or burden to the infant, with the potential for direct benefit.^27^ However, the analysis of PK‐PD data from these dose optimization studies at best only benefits other infants treated in the future, which assumes that the evidence generated is sufficient to inform policy and practice. Furthermore, pharmaceutical manufacturers may need further motivation to develop a formulation, tablet strength and fixed‐dose combination ratio suitable for use in infants and very young children.

## 4 THE ROLE OF POPULATION PK‐PD MODELLING IN ANTIMALARIAL DOSE OPTIMIZATION IN INFANTS

Population PK‐PD modelling (i.e., nonlinear mixed‐effects modelling) has the potential to overcome the challenges presented by standard dose optimization studies. Population PK‐PD models have three components: structural models that describe the time course of a quantified mean response (i.e., fixed effects), in most cases these can be drug concentrations (PK) or treatment response (PD, efficacy/safety); stochastic or statistical models that define the between‐ and within‐patient variability in the observed data (i.e., random effects); and covariate models that quantify the influence of factors that could affect the observed individual response such as age, nutritional status and disease severity.^28^ The main strength of population PK‐PD modelling is that it fits the model to data both on a population and individual level, which enables the analysis of very sparse, often unbalanced data, where each individual may contribute a small number of samples, and the number of samples/timings can vary between individuals.^28^, ^29^, ^30^ This makes it particularly relevant for use in the analysis of data from dose optimization studies in infants, where PK and PD data sampling may be sparse and collected at opportunistic time points. Additionally, PK‐PD modelling can be used to inform the design of dose optimization clinical trials to ensure adequate number and timing of sampling, and the power to detect differences in pharmacokinetic or pharmacodynamic outcomes.^31^ Thus, in optimizing the dosing of antimalarials in a subpopulation that is difficult to study, such as infants, PK‐PD modelling could be utilized as a tool for designing as well as analysing data from such studies.^32^ This would allow carefully designed and early inclusion of infants in dose optimization clinical trials, preferably early enough to inform decisions on tablet strength and ratios of fixed‐dose combination treatments (i.e., phases IIb or III). Other advantages of applying PK‐PD modelling approaches in paediatric PK studies in malaria include an improved mechanistic understanding of drug effects and the ability to investigate complex circumstances such as drug–drug interactions and disease progression effects.^21^

## 5 PREVIOUS USE OF PK‐PD MODELLING TO INFORM ANTIMALARIAL DOSE OPTIMIZATION IN INFANTS

There is generally a paucity of evidence on the use of PK‐PD modelling for antimalarial dose optimization in infants. Nevertheless, we have utilized evidence on dose optimization in children to highlight the potential role of PK‐PD modelling in antimalarial dose optimization for use in infants. Table 2 details examples of large individual patient data analyses that have used population PK‐PD modelling techniques to optimize the dosing of some of the commonly used first‐line antimalarial therapies against *Plasmodium falciparum* in children. These analyses on artesunate‐amodiaquine,^33^ AL,^13^ DP^34^ and SP,^35^ indeed, underscore the importance of the PK‐PD modelling methodologies as a tool for dose optimization and generation of evidence to inform treatment guidelines. For example, the WHO's recommendation on dosing DP in very young children was adopted from PK‐PD dose optimization work following concerns that the manufacturer's recommended dosing regimen of piperaquine resulted in lower piperaquine concentrations than needed to provide longer‐term protection after treatment of uncomplicated malaria.^5^, ^34^, ^36^, ^37^ Other examples of studies on antimalarial therapies that have applied PK‐PD techniques for malaria (severe and uncomplicated) treatment as well as chemoprevention are further reviewed in Table 3. ^38^, ^39^, ^40^, ^41^, ^42^, ^43^, ^44^, ^45^

**TABLE 2:** Individual patient data analyses using population PK and PD methodologies to optimize antimalarial dosing in children.

Antimalarial, year, country	Age range of children included in analysis	Indication for treatment	Total no. of participants (all age groups) included in the analysis	No. of participants who were children [% of total]	No. of studies included in the analysis	Drug exposure parameter assessed	PD parameter	Summary of key message(s)	Conclusions/recommendations
Amodiaquine^33^									
2018, multicountry	12 months to 5 years	Uncomplicated *P. falciparum* malaria	261	95 [36%]	6	Day 7 plasma concentrations and *C*_max_	PD assessment not conducted	Body size and age affected amodiaquine clearance Bioavailability was 22.4% lower at the start of treatment than during convalescence, suggesting malaria disease effect Assuming birth at term, clearance rates for amodiaquine and desethylamodiaquine reached 50% of adult maturation at 2.8 months	Need for optimized dosing regimens to achieve similar drug exposure in all age groups Higher doses of amodiaquine may be needed for some weight ranges (8 kg, 15–17 kg)
Lumefantrine^13^									
2018, multicountry	12 months to 10 years	Uncomplicated *P. falciparum* malaria	3486	1289 [37%]	26	Day 7 plasma concentrations	Malaria recurrence by Day 42 post treatment	Currently recommended 6‐dose regimen in children weighing <15 kg and 15–24 kg resulted in 24.2% and 13.4% lower predicted median venous lumefantrine concentrations at Day 7, respectively, when compared to adult patients	A 5‐day regimen of current weight‐based standard twice‐daily doses for small children is most favourable from a pharmacological perspective
Piperaquine^34^									
2017, multicountry	6 months to 10 years	Uncomplicated *P. falciparum* malaria	728	448 [62%]	11	Day 7 plasma concentrations	PD assessment not conducted This analysis was informed by previous pooled analysis of 26 studies that showed significant risk of recrudescent malaria in young children (1–5 years) following treatment with dihydroartemisinin‐piperaquine	Small children had a substantially lower piperaquine exposure after recommended dosing regimens Derived population pharmacokinetic model was used to develop a revised dose regimen of dihydroartemisinin‐piperaquine that is expected to provide equivalent piperaquine exposures safely in all patients, including in small children with malaria	This work was part of the evidence that informed the World Health Organization technical guidelines development group in the development of the 2015 treatment guidelines for malaria
Sulfadoxine and pyrimethamine (SP)^35^									
2018, multicountry	<2–5 years	Uncomplicated *P. falciparum* malaria	801	415 [52%]	4	Day 7 plasma concentrations	PD assessment not conducted	Underweight‐for‐age children were found to have 15.3% and 26.7% lower bioavailability of sulfadoxine and pyrimethamine. Under current dosing recommendations, the simulation predicted that the median Day 7 concentration was below the 25th percentile for a typical adult patient (50 kg) for sulfadoxine for patients in the weight bands of 8–9, 19–24 kg and 8–9, 14–24 kg for pyrimethamine	Evidence‐based dosing regimen was constructed that would achieve sulfadoxine and pyrimethamine exposures in young children and underweight‐for‐age young children that were similar to those currently seen in a typical adult

**TABLE 3:** Other examples of population pharmacokinetic‐pharmacodynamic studies to optimize dosing of antimalarial medications in children for malaria therapeutic and preventive treatment.

Antimalarial, year, country	Age range of children included in analysis	Route of administration and indication for treatment	No. of participants (all age groups) included in the study	No. of participants who were children [% of total]	Drug exposure parameter assessed	Pharmacodynamic (PD) parameter	Summary of key message(s)	Conclusions/recommendations
Quinine								
2005, Cameroon^38^	6 months to 6 years	Oral, uncomplicated *P. falciparum* malaria treatment	30	30 [100%]	Plasma concentrations on Days 1–3 with calculated clearance and volume of distribution	Parasite load reduction within 72 h Malaria recurrence on Days 7 and 14	Clearance and volume of distribution were positively correlated with body weight and increased over time The time to a 4‐log reduction of the initial level of parasitaemia was related to the average quinine concentration from 0 to 72 h	Need to evaluate the efficacy of a 5‐day treatment course in a larger clinical trial
2001, Ghana^44^	12 months to 10 years	Intramuscular loading and maintenance doses, severe *P. falciparum* malaria treatment	120	120 [100%]	Plasma concentrations from 0 to 12 h post dosing with calculated clearance and volume of distribution	Parasite clearance in 72 h	A two‐compartment model with first‐order absorption and elimination gave post hoc estimates for pharmacokinetic parameters that were consistent with those derived from non‐population pharmacokinetic studies of clearance and volume of distribution Intramuscular quinine associated with minor, local toxicity (13 of 108; 12%), and one or more episodes of postadmission hypoglycaemia in 11 patients (10%)	A loading dose of intramuscular quinine resulted in predictable population pharmacokinetic profiles in children with severe malaria and may be preferred to the intravenous route of administration in some circumstances
2013, Tanzania^39^	4 months to 8 years	Intramuscular loading and maintenance doses, severe *P. falciparum* malaria treatment	75	75 [100%]	Plasma concentrations from 0 to 24 h post dosing with calculated clearance and volume of distribution	Median time to reach a 50% reduction in hazard (survival over time)	Quinine exposure was reduced at lower body weights after standard weight‐based dosing; there was 18% less exposure over 24 h in patients weighing 5 kg than in those weighing 25 kg. Maximum plasma concentrations after the loading dose were unaffected by body weight No evidence of dose‐related drug toxicity with the loading dosing regimen	Intramuscular quinine was rapidly and reliably absorbed in children with severe falciparum malaria Based on these pharmacokinetic data, a loading dose of 20 mg salt/kg was recommended, provided that no loading dose was administered within 24 h and no routine dose was administered within 12 h of admission
Pyronaridine								
2015, multicountry^45^	6 months to 15 years	Oral, uncomplicated *P. falciparum* malaria treatment	349	349 [100%]	AUC_0–∞_ with derived clearance and volume of distribution	PD assessment not done	Age was identified as a significant predictor of pyronaridine peripheral volume of distribution Formulation was a significant covariate on pyronaridine absorption rate constant	Simulations of pyronaridine concentration–time profiles showed similar exposures across paediatric weight ranges, supporting the proposed labelling for weight‐based dosing of pyronaridine granules
Artesunate and metabolite dihydroartemisinin (DHA)								
2006, multicountry^40^	11 months to 15 years	Intrarectal, severe *P. falciparum* malariatreatment	179	[62%]	AUC_0–6h_ with derived clearance and volume of distribution	Treatment outcome Early rescue treatment Failure of baseline parasitaemia to fall 40% by 12 h Time to clear 50% and 90% of baseline parasitaemia	Gender was associated with increased mean clearance (CL/F) of DHA by 1.14 (95% CI: 0.36–1.92) (L/kg/h) for a male compared with a female Weight was positively associated with volume of distribution (V/F). Larger V/Fs were observed for the patients requiring early rescue treatment compared with the remainder, independent of any confounders No associations between the parasitological responses and the posterior individual estimates of V/F, CL/F, and AUC_0–6h_ were observed	Pharmacokinetic properties of DHA were affected only by gender and body weight Patients with the lowest area under the DHA concentration curve did not have slower parasite clearance, suggesting that rectal artesunate is well absorbed in most patients with moderately severe malaria
Mefloquine								
2006, Thailand^43^	2–15 years	Oral, uncomplicated *P. falciparum* malariatreatment	50	Not indicated	AUC_0–∞_ with derived clearance and volume of distribution	Malaria recurrence by Day 63 post dosing	AUC_0–∞_ was 40% higher than previous estimates for patients given the equivalent conventional‐dose regimen (mefloquine given as 15 mg/kg and then 10 mg/kg on the second and third days of treatment) Splitting the 25 mg/kg dose of mefloquine into three doses of 8 mg/kg each resulted in improved oral bioavailability compared to the conventional split‐dose regimen results.	New regimen expected to be well tolerated and result in equivalent therapeutic response to conventional split‐dose regimen
Piperaquine								
2021, Uganda^41^	2 months to 2 years	Oral, *P. falciparum* malaria weekly *vs*. monthly chemoprevention	280	280 [100%]	Plasma concentration associated with 95% protection from malaria	Cumulative malaria treatment hazard from 2 to 36 months of age	Compared to dihydroartemisinin‐piperaquine (DP) every 12 weeks, DP every 4 weeks is associated with 95% protective efficacy (95% CI: 84–99%). Piperaquine plasma concentration of 15.4 ng/mL reduces malaria hazard by 95%. Malnutrition reduces piperaquine exposure.	Simulated regimens showed DP every 4 weeks is optimal across a range of transmission intensities, and age‐based dosing improves malaria protection in young or malnourished children.
2019, Burkina Faso^42^	2 months to 5 years	Oral, *P. falciparum* seasonal malaria chemoprevention (SMC)	179	179 [100%]	Minimum inhibitory concentration of piperaquine during SMC with derived PK parameters (clearance and volume of distribution)	Time‐to‐malaria infection during 4 months of study period (comprising three rounds of chemoprevention and 2 months of passive follow‐up)	Increasing the DP dosage and extending the dose schedule to four monthly doses result in a predicted relative reduction in malaria incidence of up to 58% during the high transmission season	The higher and extended dosing schedule to cover the high transmission period for seasonal malaria chemoprevention could improve the preventive efficacy substantially

Notably, there is limited data on antimalarial dosing in infants under 6 months of age when antimalarial therapies are used for malaria chemopreventive treatment. Furthermore, the use of PK‐PD methods to optimize antimalarial dosing in this age group is limited (Tables 2 and 3). The lack of evidence on antimalarial dosing in infants under 6 months of age is thought to be due to the perception that malaria in this age group is rare because of maternal antibody protection that is passed on at birth.^1^, ^46^ However, this has been shown to not be the case; for example, in a large cross‐sectional study in Uganda (*n* = 7785) the malaria parasitaemia positivity rate in infants under 6 months of age was 31.6%.^1^ This calls for urgent antimalarial dosing recommendations in infants under 6 months for both uncomplicated malaria treatment and chemoprevention. Population PK‐PD methodologies have a potential role to support this and ensure accurate and safe dosing regimens in this vulnerable subpopulation.

Additionally, in most analyses reported in Tables 2 and 3, multiple antimalarial exposure parameters such as AUC, *C*_max_ and Day 7 concentrations were utilized. While this variation reflects the different primary aims of the studies that were included, it could limit comparisons between analyses and interpretations of PK‐PD associations. Since Day 7 antimalarial concentrations of long‐acting drugs such as lumefantrine are predictive of overall exposure and antimalarial efficacy by Day 28,^47^ and generally assessed in most antimalarial studies, utilizing such a standardized exposure parameter as a proxy for overall exposure could potentially overcome this limitation.

## 6 LIMITATIONS AND GAPS OF POPULATION PK‐PD MODELLING FOR DOSE OPTIMIZATION IN INFANTS

Despite being a robust technique for analysing drug exposure–response data, even when such data is sparse, population PK‐PD modelling has some limitations. First, the rapidly changing physiological states, such as those related to age in infancy or disease conditions, may render the assumptions of the structural model inaccurate or overly simplistic.^48^ Second, since infants experience age‐ or weight‐related changes, there is always a need to apply scaling techniques to adjust for these varying physiological states when estimating primary parameters of drug exposure as well as optimizing dosing regimens.^49^, ^50^ Third, when applied to data that are collected from clinical trials or observation studies, the assessment of covariates that may affect drug exposure is limited by study inclusion criteria and the data that were collected, thus studies are often underpowered to identify significant covariates.^51^, ^52^ While individual patient PK‐PD data meta‐analyses may include all available studies to ensure adequate statistical power, other covariates such as time of food intake before or after drug administration might be excluded. Additionally, the model may not comprehensively account for all possible programmatic scenarios in which the optimized dosing regimens would be deployed, such as dosing with routine health facility visits (e.g., for vaccination), or dosing without food or suboptimal adherence with doses to be taken at home (e.g., ACT dosing on Days 2 and 3). Fourth, although population PK‐PD models allow for a better understanding of the processes of drug exposure, including an assessment of compartmental pharmacokinetics, developing models that best fit the observed data can be a lengthy process,^53^ and often requires substantial skills that are only present in a few malaria‐endemic countries. This may preclude the timely availability of optimized dosing regimens for use in programmatic settings. Fifth, the development of PK‐PD models relies on accurate and sensitive drug measurements in small blood volumes,^29^ which requires expensive equipment (mass spectroscopy) and experienced staff to operate these machines. These assays also come at a high cost of US$20–100 per sample, which is often prohibitive. Furthermore, the complexity of population PK‐PD models may not always be easily understood by stakeholders, such as national malarial control programs.^54^ This may further limit the utility of population PK‐PD tools in malaria‐endemic areas to inform local optimal dosing of antimalarials.

## 7 OTHER MODELLING TECHNIQUES FOR DOSE OPTIMIZATION IN INFANTS

Physiologically based PK (PBPK) modelling is another tool that can be used to optimize the dosing of antimalarial therapy in infants.^55^, ^56^, ^57^ PBPK models account for the development of organs and the ontogeny of specific enzymes, such as cytochrome P450, and transporters that determine the age‐related pharmacokinetics of various therapies.^58^ This enables them to provide a clear mechanistic understanding of the processes behind altered drug exposure. As a result, they have been previously applied to provide a mechanistic understanding of drug disposition in infants and guide optimal dosing in various complex scenarios of both antimicrobial therapy and other therapeutic agents.^50^, ^59^, ^60^, ^61^, ^62^ Additionally, they have been applied in drug regulatory reviews^63^ and are being used to inform the design of paediatric drug development studies.^64^ Nevertheless, these PBPK models, unlike population PK‐PD models, often describe average persons in populations and are not able to dissect inter‐individual or unexplained variability. Furthermore, PBPK models apply assumptions of rates of individual processes of absorption, distribution, metabolism and excretion, and these may not always be known for each antimalarial medication of interest.^65^ This makes it challenging to easily apply them in practice. Nonetheless, the mechanistic insights that they offer could be capitalized on when combined with PK‐PD models to optimize dosing in infants. However, their inherent complexity makes them computationally costly and difficult to use.^66^

Recently, machine learning (ML) algorithms have been identified as potential platforms to identify doses for antimicrobial agents. Indeed, ML PK‐PD models have been shown to accurately predict concentrations of rifampicin in the treatment of tuberculosis^67^ and beta‐lactams when managing hospital‐acquired and ventilator‐associated pneumonia.^68^ Nonetheless, the main limitation of ML PK‐PD modelling is that it may not offer a mechanistic understanding of underlying assumptions of drug exposure. Their application for use in infants and other complex subpopulations needs further exploration.

## 8 CONCLUSION

PK‐PD modelling has a potentially unique advantage of using sparse unbalanced data to inform antimalarial dose optimization in infants, who bear a disproportionately high burden of malaria but are usually excluded from clinical trials on antimalarials. Nevertheless, it may not account for all physiological, clinical and programmatic scenarios in which these preventive treatments and uncomplicated malaria treatments would be used. Thus, other modelling tools, such as PBPK modelling techniques, could further strengthen the ability of PK‐PD models to provide a quicker understanding of mechanistic processes related to drug exposure in infants. To utilize modelling well, there is a need to generate reasonably minimal, but useful, PK and PD data in this subpopulation, especially in children under 6 months of age. This should be done as early as ethically possible in antimalarial drug development to inform their optimal dosing.

### 8.1 Nomenclature of targets and ligands

Key protein targets and ligands in this article are hyperlinked to corresponding entries in http://www.guidetopharmacology.org, and are permanently archived in the Concise Guide to PHARMACOLOGY 2023/24.^69^

## AUTHOR CONTRIBUTIONS

**Clifford G. Banda:** Conceptualization; writing—original draft; writing—review & editing. **Joel Tarning:** Conceptualization; writing—review & editing. **Karen I. Barnes:** Conceptualization; Writing—review & editing.

## CONFLICT OF INTEREST STATEMENT

All authors declare no conflicts of interest.

## Data Availability

No data are associated with this article.
